# Development of myocarditis and pericarditis after COVID‐19 vaccination in children and adolescents: A systematic review

**DOI:** 10.1002/clc.23965

**Published:** 2023-01-02

**Authors:** Maurish Fatima, Muhammad H. A. Khan, Muhammad S. Ali, Muhammad Osama, Huzaifa A. Cheema, Aleena Ahmed, Amna Nisar, Muhammad W. Murad, Hareem Farooq, Muhammad A. U. Rehman, Sarya Swed, Usman A. Akbar

**Affiliations:** ^1^ Department of Medicine King Edward Medical University Lahore Punjab Pakistan; ^2^ Department of Medicine Shanxi Medical University Yuci District Jin Zhong City Shanxi province China; ^3^ Faculty of Medicine Aleppo University Aleppo Syria; ^4^ Division of Infectious Diseases University of Louisville Louisville Kentucky USA

**Keywords:** adolescents, children, COVID‐19 vaccination, myocarditis, pericarditis

## Abstract

Myocarditis and pericarditis have been reported after COVID‐19 vaccine administration in children and adolescents, raising the concern about their possible association with these vaccines. The objective was to explore the incidence, clinical presentation, and association of myocarditis and pericarditis with COVID‐19 vaccines in children and adolescents. We conducted a systematic literature search on three databases, that is, Cochrane, MEDLINE/PubMed, and EMBASE from inception till March 2022. A total of three case reports, four case series, and six observational studies were included in the review. For case reports and case series, the mean age of the patients was 17.4 years, with 96.9% being male. Chest pain (*n* = 31, 93.9%), fever (*n* = 18, 54.5%), myalgias (*n* = 15, 45.4%) and headache (*n* = 9, 27.2%) were the most common presentations. Out of 33 patients, 32 (96.9%) of patients received Pfizer‐BioNTech whereas only one (3.03%) received Moderna (mRNA 1273). Clinical investigations revealed ST elevation (*n* = 32, 97%), and elevated CRP (*n* = 9, 27.2%) and cardiac troponin (*n* = 29, 87.8%). The pooled incidence of myocarditis and pericarditis from observational studies was (0.00063%) and (0.000074%) %, respectively. Myocarditis and pericarditis in children and adolescents after the COVID‐19 vaccines were more prevalent among males and more commonly observed after the second dose of Pfizer. Though the overall incidence was low, however, the clinicians should consider myocarditis and pericarditis as probable diagnosis when encountering young patients, with a history of vaccine administration, presenting with suggestive findings.

## 1 INTRODUCTION

COVID‐19 was initially reported in December 2019 in Wuhan, China, and over 317 million global cases of COVID‐19 have been reported ever since.^1^ To deal with this public health emergency of international concern (PHEIC), numerous expedited vaccination trials were conducted, which eventually led to the successful development of antiviral vaccines. The different types of coronavirus vaccines include mRNA‐based vaccines (i.e., Pfizer‐BioNTech, Moderna, Comirnaty), recombinant adenoviral vector vaccines (i.e., Johnson & Johnson/Janssen, Oxford‐AstraZeneca, and Sputnik V), and the inactivated whole viral vaccines (i.e., Sinovac Biotech and Sinopharm).^2^ Mass immunization campaigns are currently underway across the globe, and our understanding of the virus, as well as the vaccine, is improving. More than 10 million doses of vaccines have been administered across the world and published literature has reported a significant reduction in severity of COVID‐19 infection, hospitalization, and mortality rates.^3^ Postmarketing surveillance (Phase 4 trials) is an integral way to understand the various side effects of different types of vaccines, especially in case of expedited approvals. The results of Phases 2 and 3 trials have revealed various adverse effects of COVID‐19 vaccines which range from mild fever, fatigue, headache, muscle pain, and diarrhea to serious adverse effects such as myocarditis, pericarditis, thrombocytopenia, lymphadenopathy, bell's palsy, and cerebrovascular accident.^4^ Among these adverse effects, several cases of myocarditis and pericarditis following COVID‐19 vaccine administration have also been reported around the world.^5^

As of April 21, many cases of myocarditis and pericarditis in children and adolescents have been reported after administration of mRNA COVID‐19 vaccine, however, most of the cases were mild and self‐resolving with rare instances of hospital admission.^6^ Myocarditis and pericarditis refers to the inflammation of myocardium and pericardium of heart respectively and commonly occurs as a consequence of viral infection.^7^, ^8^, ^9^ It is usually a self‐limited condition that responds to conservative management without any long‐term sequelae but complications such as cardiomyopathy and heart failure have been reported.^10^

The incidence of myocarditis in children is usually very low; accounting for 0.7% as reported in a large retrospective study.^11^ Whereas, the incidence of myocarditis in adults is 1.5 million cases worldwide per year.^12^ Viral infection has been reported to be the most common cause of myocarditis in children.^13^, ^14^ In the past, myocarditis has been reported as a side effect of live attenuated vaccines such as smallpox and influenza vaccines in children and adolescents.^15^ The reporting of COVID‐19 vaccine‐related myocarditis and pericarditis cases are being investigated by safety agencies including the Centre of Disease Control and Prevention (CDC) in the United States and Pharmacovigilance Risk Assessment Committee (PRAC) in Europe.^16^ Several published age, gender, and vaccine type‐based analyses have reported an increased risk among young males following mRNA vaccines such as Pfizer‐BioNTech and Moderna.^17^ So far, it is still unclear if these results really reflect an increase in incidence or simply better reporting and recollection bias. The aim of this systematic review is to explore the incidence, clinical presentation, management, and association of myocarditis and pericarditis with the COVID‐19 vaccines in children and adolescents. To the best of our knowledge, this is the first systematic review on this topic with the aim of providing a comprehensive outline of available evidence regarding COVID‐19 vaccine‐associated myocarditis and pericarditis.

## 2 METHODS

The review has been registered on The International Prospective Register of Systematic Reviews (PROSPERO CRD 42021282961). The study was performed according to the Preferred Reporting Items for Systematic Reviews (PRISMA) guidelines.^18^ A systematic literature search was conducted on the following three databases: Pubmed/MEDLINE, Cochrane, and EMBASE from inception till March 2022. No filter in terms of time, study design, language, country of publication, and so forth. was used to retrieve all the available literature. The complete search string for PubMed is given in Supporting Information: Table S1.

We considered only those studies which included the population of children and adolescents (from birth up to 19 years of age) who had received their first or second dose of COVID‐19 vaccine and had developed either myocarditis or pericarditis. Review articles, editorials, and those original articles that reported other side effects of vaccination but did not discuss myocarditis and pericarditis specifically, and articles in languages other than English were excluded from this review.

**Figure 1:**
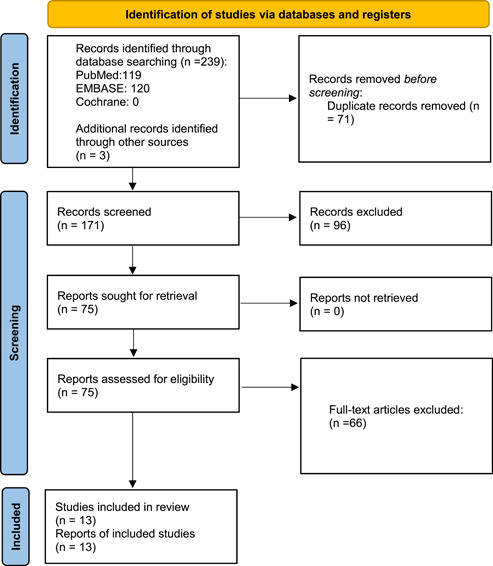
PRISMA flow chart. PRISMA, Preferred Reporting Items for Systematic Reviews.

The search of three databases identified 242 articles. A total of 171 articles were removed due to duplication, and 96 articles were excluded due to irrelevance to the topic (Figure 1). After rigorous screening, 12 articles comprising three case series, four case reports, and six original studies were included in our review.^19^, ^20^, ^21^, ^22^, ^23^, ^24^, ^25^, ^26^, ^27^, ^28^, ^29^, ^30^, ^31^ Identified studies were uploaded on Mendeley, and duplicates were removed. Initially, the articles were screened on the basis of title and abstract, after which full articles were reviewed. Articles were searched and extracted by two reviewers (M. F. and H. A. C) and a third investigator (M. H. A. K) was contacted to resolve any discrepancies.

Continuous variables were presented as means ± standard deviations, and categorical variables were presented as absolute values and percentages. Microsoft Excel was used to extract data and do calculations. Mendeley was used to add the references. The retrieved results of the case reports and case series are summarized in the form of two tables (Tables 1 and 2). One table focuses on the demographics, medical history, and outcomes, whereas the second is based on relevant medical investigations and diagnostic findings. The summary of included original articles (observational studies) has been delineated in Table 3. Table 3 consists of columns of study design, author and year of publication, country, sample size, age (range), gender, follow‐up, comparator group (if any), experimental group characteristics, outcomes (myocarditis or pericarditis or both), clinical features of the reported cases, and results (incidence, incidence rate ratio [IRR], cumulative incidence, risk difference [RD], rate ratio depending on data reported by respective study).

**Table 1:** Demographics of patients with myocarditis and pericarditis after COVID‐19 vaccine

Sr. No.	Doma in	References	Country reported	Number of patients		Age (years), gender (M/F)	Medical history	Type of vaccine administered	Myocarditis/pericarditis	Time between vaccine administration and development of myocarditis/pericarditis
1	Case series	Marshall et al.^27^	USA	7	Patient no. 1	16, M	Not significant	Pfizer‐BioNTech	Myocarditis	2 days after second dose
					Patient no. 2	19, M	Not significant	Pfizer‐BioNTech	Myocarditis	3 days after second dose
					Patient no. 3	17, M	Not significant	Pfizer‐BioNTech	Myopericarditis	2 days after second dose
					Patient no. 4	18, M	Not significant	Pfizer‐BioNTech	Myocarditis	3 days after second dose
					Patient no. 5	17, M	Not significant	Pfizer‐BioNTech	Myocarditis	3 days after second dose
					Patient no. 6	16, M	Not significant	Pfizer‐BioNTech	Myocarditis	3 days after second dose
					Patient no. 7	14, M	Not significant	Pfizer‐BioNTech	Myopericarditis	2 days after second dose
2	Case report	Minocha et al.^30^	USA	1		17, M	Not significant	Pfizer‐BioNTech	Myocarditis	2 days after second dose
3	Case series	Dionne et al.^25^	USA	15		12–18 (median = 15 years) 0.14 out of 15 were male	Not significant	Pfizer‐BioNTech	Myocarditis	1 to 6 days after the second dose of the vaccine in all but 1 case
4	Case series	Dickey et al.^24^	USA	6	Patient no. 1	35–40, M	Not significant	Pfizer‐BioNTech	Myocarditis	4 days after second dose
					Patient no. 2	16–20, M	Not significant	Pfizer‐BioNTech	Myocarditis	3 days after second dose
					Patient no. 3	20–25, M	Not significant	Moderna	Myocarditis	4 days after second dose
					Patient no. 4	20–25, M	Not significant	Pfizer‐BioNTech	Myocarditis	2 days after second dose
					Patient no. 5	16–20, M	Not significant	Pfizer‐BioNTech	Myocarditis	4 days after second dose
					Patient no. 6	16–20, M	Not significant	Pfizer‐BioNTech	Myocarditis	3 days after second dose
5	Case report	Isaak et al.^26^	USA	1		15, M	Not significant	Pfizer‐BioNTech	Myocarditis	1 day after second dose
6	Case report	Watkins et al.^28^	USA	1		20, M	Tobacco+, COVID+ history	Pfizer‐BioNTech	Myocarditis	2 days after second dose
7	Case series	Park et al.^29^	USA	2	Patient no. 1	15, M	Not significant	Pfizer‐BioNTech	Myocarditis	3 days after first dose
					Patient no. 2	16, M	Not significant	Pfizer‐BioNTech	Myocarditis	2 days after second dose

**Table 2:** Clinical presentation, lab investigations, and diagnostic findings in patients with myocarditis and pericarditis after COVID‐19 vaccine

Sr. no.	Doma in	References	Clinical features	ECG findings	Lab investigations	Treatment	Echocardiogram findings	Diagnostic criteria (CMR imaging)	Additional comments
1	Case series	Marshall et al.^27^	Fatigue, poor appetite, fever of 38.3°C, and pain in the chest and both arms.	Atrioventric ular dissociation with junctional escape and ST elevation	CRP = 12.3 mg/L, Troponin I = 2.59 ng/ml	IVIg, IV methylprednisolone, oral prednisone, IV ketorolac	Normal	CMRI demonstrated apical and midchamber lateral wall subepicardial LGE.	Recovered and discharged
			Acute, persistent chest pain, myalgias, fatigue, weakness, and subjective low‐fevers grade	Diffuse ST elevation	Troponin T = 232 ng/L, CRP = 6.7 mg/dl	IV ketorolac, colchicine, Aspirin	Echocardiogram was normal	CMR showed patchy, midwall LGE along the basal inferolateral wall segment.	Recovered and discharged
			Chest pain. It worsened when lying flat and was associated with left arm pain and paresthesias	T wave abnormalities with diffuse ST elevation	Troponin I = 5.550 ng/ml, CRP = 25.3 mg/L	Ibuprofen	Normal	CMRI showed delayed enhancement at the LV subepicardial basal anterolateral segment and basal to mid‐ventricular inferolateral segments, consistent with myocardial necrosis, evidence of diffuse fibrosis on T1 weighted imaging, and myocardial edema on T2 mapping.	Recovered and discharged
			Chest pain, malaise, arthralgia, myalgia, and subjective fever. It worsened when lying flat and was associated with left arm pain and paresthesias	ST elevation	Troponin T = 1.09 ng/ml, CRP = 12.7 mg/dl	IVIg, methylprednisolone, oral prednisone, Ibuprofen, Aspirin	Normal	CMRI demonstrated edema, hyperemia, and fibrosis.	Recovered and discharged
			Chest pain, sore throat, headache, dry cough, and body aches. He also developed midsternal chest pain that was worse when lying flat and radiated to the left arm	ST‐elevation	Troponin T = 3.21 ng/ml, CRP = 18.1 mg/dl	IVIg. IV methylprednisolone, oral prednisone, Ibuprofen, Aspirin	Normal	CMRI demonstrated diffuse, nearly complete transmural LV free wall gadolinium enhancement.	Recovered and discharged
			Midsternal Chest Pain, malaise, and subjective fever	ST‐segment elevation	Troponin T = 0.01 ng/ml, CRP = 1.8 mg/dl	IVIg, oral prednisone	Normal	LGE, diffuse myocardial edema.	Recovered and discharged
			Pleuritic chest pain and shortness of breath	ST‐segment elevation	CRP = 12.7 mg/dl, Troponin I = 0.02 ng/ml	NSAID, famotidine, furosemide	Echocardiogram showed mildly depressed left and right ventricular systolic	LGE (subepicardial) involving mid and apical LV free wall, myocardial edema, hyperemia.	Recovered and discharged
2	Case report	Minocha et al.^30^	Sudden onset of severe, burning left‐sided chest pain that radiated to the left shoulder and the upper left arm. He reported that the chest pain worsened with exertion and movement	Diffuse ST‐segment elevations	Troponin = 2.3 ng/ml, CRP = 29 mg/L	NSAIDs	Not mentioned	CMR showed low normal LVEF (53%), trivial pericardial effusion, and subepicardial lGE.	Recovered and discharged
3	Case series	Dionne et al.^25^	Chest pain in all fever in 10 patients, myalgia in 8 patients, and headache in 6 patients.	Diffuse ST‐segment elevation present on admission in six patients and at some time during hospital admission in eight patients. Four patients had nonspecific ST‐segment changes. One patient had nonsustained ventricular tachycardia during hospital admission.	Troponin levels were elevated in all patients at admission (median, 0.25 ng/ml [range, 0.08– 3.15 ng/ml]) and peaked 0.1–2.3 days after admission.	Seven patients were treated with IVIg and methylprednisolone (1 mg/kg/dose twice a day, transitioned to prednisone at time of discharge)	Three patients had global LV systolic ventricular dysfunction (EF 44%, 49%, and 53%), one of whom also had regional wall motion abnormality at the apex. Two patients with systolic dysfunction had abnormal diastolic function indices, and one patient with borderline EF (55%) had evidence of diastolic dysfunction. Five patients had abnormal global longitudinal or global circumferential strain.	LGE = 12 patients, Systolic LV dysfunction = 3 patients, Findings consistent with myocarditis = 13 patients.	Recovered and discharged
4	Case series	Dickey et al.^24^	Positional and pleuritic chest and neck pain; chills; and myalgias	Sinus rhythm with inferolateral ST‐elevation	Troponin I (ng/ml) = 5.41	Not mentioned	LVEF = 45%	Increased T2 signal and LGE in the midwall of the lateral segments in a patient who received their second SARS‐CoV‐2 vaccination 5 days earlier	Recovered and discharged
			Pleuritic and positional chest pain; rhinorrhea; headache, fever	Sinus rhythm with diffuse ST‐elevation	Troponin I (ng/ml) = 38.3	Not mentioned	LVEF = 53%	Increased T2 signal and LGE in the midwall and subepicardial layer throughout the left ventricle) in a patient who received their second SARS‐CoV‐2 vaccination 7 days earlier.	Recovered and discharged
			Pleuritic and positional chest pain; chills; myalgias; and subjective fever	Sinus rhythm with diffuse ST‐elevation	Troponin I (ng/ml) = 18.94	Not mentioned	LVEF = 58%	Increased T2 signal and LGE in the midwall and subepicardial layer of the mid‐posterolateral segment in a patient who received their second SARS‐CoV‐2 vaccination 6 days earlier.	Recovered and discharged
			Nonpositional chest pain radiating to back; myalgia; malaise, fever	Sinus rhythm with diffuse ST‐elevation and PR depression; nonsustained ventricular tachycardia	Troponin I (ng/ml) = 13.4	Not mentioned	LVEF = 48%	Not mentioned.	Recovered and discharged
			Pleuritic and positional chest pain; headache	Sinus rhythm with nonspecific T wave abnormalities	Troponin I (ng/ml) = 5.21	Not mentioned	LVEF = 46%	Not mentioned.	Recovered and discharged
			Nonpositional chest pressure; myalgias	Ectopic atrial rhythm with diffuse ST‐elevation and PR depression	Troponin I (ng/ml) = 19.7	Not mentioned	LVEF = 50%	Increased T2 signal and LGE in the subepicardial apical and apical lateral segments.	Recovered and discharged
5	Case report	Isaak et al.^26^	Fever, myalgia, and intermittent tachycardia	ST‐segment elevation in the left precordial lead	High‐sensitive cardiac troponin and C‐reactive protein levels were elevated (values not mentioned)	Not mentioned	Normal	Cardiac MRI at 1.5 T showed a normal LV size, a normal LVEF, and a small pericardial effusion. T2‐weighted short inversion time inversion recovery sequences displayed focal myocardial edema involving the lateral wall, most emphasized in the basal inferolateral segment.	Recovered and discharged
6	Case report	Watkins et al.^28^	Presented with midsternal chest pain that radiated to the left side, mild shortness of breath.	Diffuse concave ST segment elevations with PR depressions.	Troponin = 89 ng/L	Colchicine. Metoprolol, ibuprofen	LVEF = 59%	Bedside ultrasound revealed a small pericardial effusion without evidence of tamponade, which supported the diagnosis. CMR was positive for myocarditis.	Recovered and discharged
7	Case series	Park et al.^29^	Presented with acute onset, mid‐sternal, nonradiating chest pain associated with chest tightness	ST‐elevation and T wave inversion in lateral leads	Troponin T = 304 ng/L, CRP = 18.5 mg/L	Not mentioned	LVEF = 55%–60%, with basal inferior and basal inferolateral hypokinesis	CMR revealed LGE involving the basal inferior, basal to mid inferolateral, mid anterolateral, apical lateral, apical septal, and apical inferior wall segments in a subepicardial distribution pattern, consistent with myocarditis.	Recovered and discharged
			Presented with acute onset, mid‐sternal, nonradiating chest pain associated with chest tightness	ST‐segment elevation in inferolateral leads, T wave inversion	Troponin T = 431 ng/L, CRP = 24.3 mg/L	Intravenous Ig	LVEF of 45% with moderate hypokinesis of the apex and apical septum	Lab findings.	Recovered and discharged

Abbreviations: CMRI, cardiac magnetic resonance imaging; CRP, C‐reactive Protein; ECG, electrocardiogram; IV, intravenous; IVIg, intravenous immunoglobulins; LGE, late gadolinium enhancements; LVEF, left ventricular ejection fraction; NSAIDs, nonsteroidal anti‐inflammatory drugs.

**Table 3:** Characteristics of included cohort studies

Sr. No.	Study design	References	Country reported	Sample size	Age (years)	Gender (M/F)	Follow‐up	Comparator	Experimental group characteristics (vaccine administered)	Outcome	Clinical features	Results
1	Cohort study	Chua et al.^19^	Hong Kong	178 163	12–17	(89 806/88 357)	4 months	N/A	Conmirnaty = 305 406 doses administered to 178 163 individuals	Myocarditis = 16 Pericarditis = 2 Perimyocarditi s = 15 Total = 33 After second dose = 27 After first dose = 6	Chest pain = 33 Normal ECG = 6 Normal echocardiogram = 25 Normal CMRI = 7 Mild disease = 33	Incidence rate; myocarditis/pericarditis = 18.52 (95% CI, 11.67–29.09) per 100 000 persons For first dose = 3.37 (95% CI, 1.12–9.51) per 1000 persons Second dose = 21.22 (95% CI, 13.78– 32.28) per 100 000 persons Male = 32.29 (22.78–45.4) per 1000 persons Female = 4.53 (1.76–11.11)
2	Cohort study	Lai et al.^31^	Hong Kong, China	252 399	12–18	(138 319/136 565)	28 days	Unvaccinated *N* = 136 743 M/F = 68 747/67 996 First dose cohort: *N* = 136 743 Age: 14.15 ± 1.816 12 years = 28 907 13 years = 32 884 14 years = 23 134 15 years = 19 863 16 years = 11 674 17 years = 12 154 18 years = 8127 Second dose cohort: *N* = 118,300 Age: 14.426 ± 1.799 12 years = 18 235 13 years = 25 355 14 years = 22 885 15 years = 19 871 16 years = 11 677 17 years = 12 153 18 years = 8124	Vaccinated: N = 138 141 M/F = 69 572/68 569 First dose cohort: *N* = 138 141 Age: 14.17 ± 1.821 12 years = 28 008 13 years = 32 040 14 years = 23 324 15 years = 20 044 16 years = 11 951 17 years = 12 443 18 years = 8331 Second dose cohort: *N* = 119 664 Age: 14.440 ± 1.803 12 years = 18 300 13 years = 25 461 14 years = 23 091 15 years = 20 063 16 years = 11 954 17 years = 12 455 18 years = 8340 Vaccine administered: Pfizer‐BioNtech	Total cases of Myocarditis: Vaccinated: (*n* = 38, 0.02%) Unvaccinated: (*n* = 2, 0.001%) After first dose: Vaccinated: (*n* = 8, 0.005%) Unvaccinated: (*n* = 1, 0.0007%) After second dose: Vaccinated: (*n* = 30, 0.02%) Unvaccinated: (*n* = 1, 0.0008%)	N/A	Overall Incidence rate of Myocarditis: IRR for first dose cohort: 9.15, 95% CI 1.14–73.16, *p* = 0.037) IRR for second dose cohort: 29.61 (95% CI 4.04–217.07, *p* = .0009) Incidence of myocarditis: For first dose: vaccinated = 2.91 (95% CI 1.26–5.73, *p* = .03) per 100 000 Persons unvaccinated = 0.35 (95% CI; 0.01–2.03) For second dose dose Vaccinated: 12.61 (95% CI 8.51–18.00) per 100 000 vaccinated persons Unvaccinated: 0.42 (95% CI 0.01–2.34) among the unvaccinated.
3	Cohort study	Nygaard et al.^21^	Denmark	261 334	12–17	133 477/127 857	N/A	N/A	Vaccine administered: Pfizer‐BioNtech First dose: 261 334 Second dose: Not mentioned	Total cases: *N* = 15 Male = (*n* = 13, 87%) Female = (*n* = 2, 13%) 12 (80%) patients had myocarditis(*n* = 10) or myopericarditis (*n* = 2) including 1 meeting the criteria for MIS‐C after vaccination. Pericarditis: (*n* = 3, 20%)	Chest pain: (*n* = 15, 100%) Fever: (*n* = 13, 73%) Elevated CRP: (*n* = 12, 80%) Abnormal ECG: (*n* = 9, 60%) Abnormal CMRI: (*n* = 7, 46%)	The incidence of myopericarditis: 97 males and 16 females per million equaling 1 of 10 000 males and 1 in 63 000 females.
4	Descriptive study	Oster et al.^20^	USA	192 405 448	16–31	N/A	N/A	N/A	m‐RNA vaccine dose administered: 354 100 845 Time period between development of symptoms, median (IQR): 2 (1–3)	Myocarditis: 1626 Pericarditis: 684 Myocarditis: BNT162b2 m‐RNA: Male = 1109 Female = 269 After dose 1 = 216 After dose 2 = 1066 After unknown dose = 103 m‐RNA 1273: Male = 447 Female = 156 After dose 1 = 174 After dose 2 = 390 Unknown Ddse = 42 Less than 30 years that met the criteria of diagnosis of myocarditis: Male = 1050 Female = 145 Greater than 30 years: Male = 284 Female = 146 Pericarditis: BNT162b2 m‐RNA: Male = 253 Female = 163 After dose 1 = 111 After dose 2 = 240 After unknown dose = 68 m‐RNA 1273: Male = 155 Female = 107 After dose 1 = 82 After dose 2 = 134 Unknown dose = 49 Less than 30 years pericarditis: *N* = 148 Greater than 30 years: *N* = 536	Chest pain = 727/817(89%) Shortness of breath 242/817; (30%) Elevated Troponin levels: 792/809, 98% Abnormal ECG: 569/794, 71% Decrease LVEF: 84/721, 11% Abnormal CMRI: 223/312, 71%	Males comprised 82% (1334/1625) of the myocarditis case. Males aged 12–15 years = 70.7 (95% CI, 61.68–81.11) per million doses of the BNT162b2 vaccine. Males aged 16–17 years = 105.9 (95% CI, 91.65–122.27) per million doses of the BNT162b2 vaccine. Male aged 18–24 years = 52.4 (95% CI, 45.56–60.33) and 56.3 (95% CI, 47.08–67.34) per million doses of the BNT162b2 vaccine and the mRNA 1273 vaccine, respectively.
5	Retrospective Cohort	Witberg et al.^22^	Israel	2 558 421	16–≥30	1 248 433/1 309 988	42 days	N/A	BNT162b2(Pfizer/BioNTech) = 2 558 421, all of the participants received first dose whereas 2 401 605 participants received second dose.	Total cases of myocarditis = 54 cases of myocarditis by sex and age: Male sex = 51 Female sex = 3 Either sex, 16–29 years = 32 Either sex ≥ 30 years = 22 Male, 16–29 years = 31 Female, 16–29 years = 1 Male ≥30 years = 20 Female ≥30 years = 2	Mild myocarditis = 41(76%) Intermediate myocarditis = 12 (22%) LVD = 14(26%) chest pain = 44 (81%) Dyspnea = 3 (5.5%) Fever = 5 (9%) Pericardial effusion = 10 (18.5%) ECG changes = 38 (70%) Elevated Troponin T = 41	Cumulative Incidence (95% CI) for all cases of myocarditis: All vaccinated participants: 2.13 (1.6–2.70) Male sex = 4.12 (2.99–5.26) Female sex = 0.23 (0–0.49) Either sex, 16–29 years = 5.49 (3.59–7.39) Either sex ≥30 years = 1.13 (0.66–1.60) Male, 16–29 years = 10.69 (6.93–14.46) Female, 16–29 years = 0.34 (0–1) Male ≥30 years = 2.11 (1.19–3.04) Female ≥30 years = 0.20 (0–0.48)
6	Retrospective cohort study	Mevorach et al.^23^	Israel	9 289 765	16–50	2 668 894/2 773 802	183 days	N/A	BNT162b2 (Pfizer/BionTech) = 5 442 696. First dose: 5 442 696, second dose: 5 125 635	Myocarditis: 136, After first dose: 19 After second dose: 117 Male recipients: 16–19 years = 3 20–24 years = 5 25–29 years = 3 30–39 years=2 40–49 years=3 ≥50 years = 1 Female recipients: 16–19 years = 0 20–24 years = 0 25–29 years = 0 30–39 years = 0 40–49 years = 1 ≥50 years = 1	Mild myocarditis: 129 (90.9%) cases Chest pain = 129 (95%) Fever = 63 (46.7%) Dyspnea = 17 (12.5%) ECG changes = 93 (68%) Elevated Troponin I or T = 136 (100%) Elevated C‐reactive protein = 118 (86.7%) LGE = 48 (35%)	Risk of myocarditis per 100 000 persons by age: Male recipients: 16–19 years = 1.34 20–24 years = 1.91 25–29 years = 1.22 30–39 years = 0.41 40–49 years = 0.65 ≥50 years = 0.10 Female recipients: 16–19 years = 0 20–24 years = 0 25–29 years = 0 30–39 years = 0 40‐49 years = 0.21 ≥50 years = 0.09 RD (95% CI) for myocarditis according to age and sex (21 days after first dose): 3.19 (2.37–4.02) Standardized Incidence ratio for myocarditis according to age, sex and dose: 5.34 (4.48–6.40), rate ratio of myocarditis within 30 days after second dose as compared to unvaccinated patients: 2.35 (1.10–5.02)

Abbreviations: CMRI, cardiac magnetic resonance imaging; ECG, electrocardiogram; F, female; IRR, incidence rate ratio; LVEF, left ventricular ejection fraction; M, male; MIS‐C, multiple inflammatory syndrome in children; RD, risk difference.

The quality of the included case series and case reports was assessed by Joanna Briggs Institute Critical Appraisal Tool.^32^, ^33^ New‐Castle Ottawa scale was employed for the methodological assessment of observational studies.^34^ Three reviewers (M. F., H. A. C., M. H. A. K.) first independently scored each article and then awarded a consensus score to each. The score reports are provided in Supporting Information: Tables S2–S4. Due to inconsistency and diversity in the reporting of outcomes, study design, and participant selection in the original articles, the results have been compiled in a qualitative manner, and a meta‐analysis was not conducted.

## 3 RESULTS

### 3.1 Case reports and case series

The data of 33 patients have been described in the case series and case reports (Tables 1 and 2). The mean age of the patients was 17.4 years (range 12–23 years). Thirty‐two (96.9%) patients were male whereas only 1 (3.03%) patient was female. Only one patient had a previous history of SARS‐CoV‐2 infection. Out of 33 patients, 32 (96.9%) of patients received Pfizer‐BioNTech whereas only 1 patient received Moderna (mRNA 1273). 93.9% (*n* = 31) had myocarditis following the COVID‐19 vaccine and two (6%) patients were diagnosed with myopericarditis. A total of 31 (93.9%) patients developed myocarditis or myopericarditis after the second dose and 2 (6%) developed the symptoms after the first dose. The mean time period between the development of symptoms and vaccine administration was 2.6 days for 18 patients and between 1 and 6 days for the 15 patients in a case series where individual data has not been presented.

In terms of clinical presentation, chest pain (*n* = 31, 93.9%), fever (*n* = 18, 54.5%), myalgias (*n* = 15, 45.4%), and headache (*n* = 9, 27.2%) were leading complaints. The treatment included intravenous immunoglobulins (*n* = 12, 36.4%), intravenous methylprednisolone (*n* = 10, 30.3%), oral prednisone (*n* = 11, 33.3%), and ibuprofen (*n* = 4, 12.1%) mainly. C‐reactive protein (CRP) values were reported in 11 (33.3%) patients out of which 9 (27.2%) patients had values greater than normal (normal < 10 mg/dl) and 29 (87.8%) patients presented with elevated serum troponin levels (normal range Troponin I = 0–0.04 ng/ml, Troponin T = 14–30 ng/ml). A total of 32 (97%) patients presented with ST‐segment elevation in ECG (electrocardiography) findings, 22 (66.7%) patients had normal left ventricular ejection fraction (LVEF) (range normal: 55–70, low normal: 50–55) and 11 (33.3%) patients had mildly reduced LVEF. Cardiac magnetic resonance (CMR) imaging findings were consistent with myocarditis in 30 (91%) patients.

### 3.2 Observational studies

A total of 204 945 530 participants were included in the observational studies, with the greatest number of participants from the United States (*n* = 192 405 448, 99.6%) (Table 3). Out of the 8 715 498 participants included in five observational studies where baseline characteristics of participants according to gender type was available, 49.1% (*n* = 4 278 929) participants were male and 50.9% (*n* = 4 436 569) were female. Most of the participants were given mRNA vaccine (*n* = 200 806 040, 97.9%). Comirnaty (COVID‐19 vaccine) was administered to 178 163 (0.086%) participants

**Figure 2:**
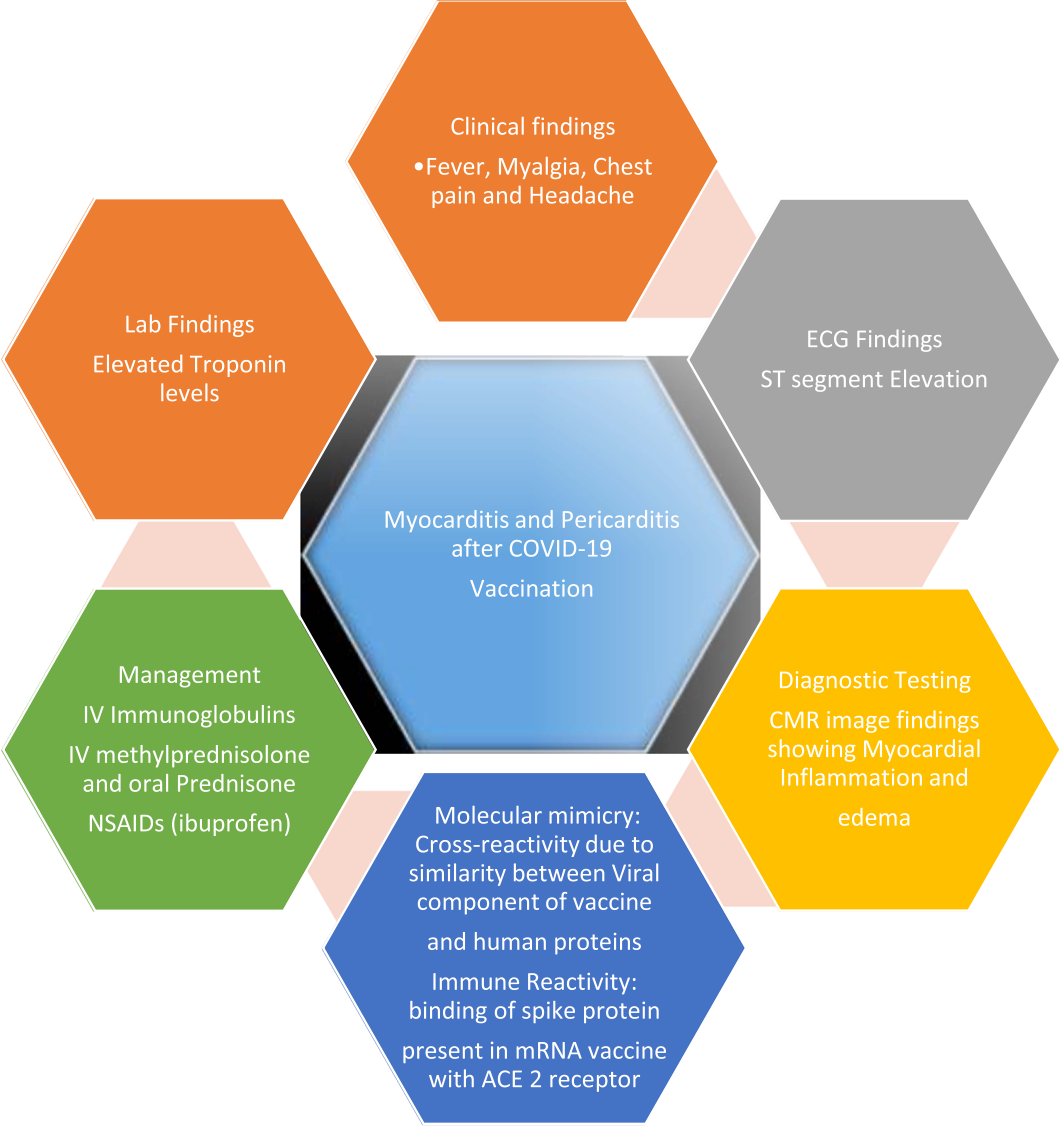
Summary of myocarditis and pericarditis after COVID‐19 vaccination

A total of 2586 (0.0012%) participants reported outcomes out of which 1464 (0.00071%) fell under the category of ≤29 years age group. Out of these 1464 patients (≤29 years) 1294 (0.00063%) developed myocarditis, 153(0.000074%) developed pericarditis and 17 (0.0000083%) presented with myopericarditis. Overall, most of the patients reported myocarditis (*n* = 1880, 0.00092%). The remaining developed pericarditis (*n* = 689, 0.00036%) and myopericarditis or perimyocarditis (*n* = 17, 0.0000083%). Out of 2586 patients, majority presented with chest pain (*n* = 948, 36.6%) and shortness of breath (*n* = 263, 10.1%). On investigations, 736 (28.4%) patients showed abnormal ECGs, 969 (37.4%) showed elevated troponin levels, and 256 (9.8%) presented with abnormal CMRI. The mean follow‐up period of four studies was 94 days. Only one study included a controlled arm, that is, an unvaccinated group. In the study by Lai et al., the overall incidence of myocarditis was higher for the vaccinated group as compared to unvaccinated group for both the first dose and second dose cohort, and there was a statistically significant risk of myocarditis with BNT162b2, especially after the second dose (first‐dose cohort: IRR = 9.15, 95% confidence interval [CI] 1.14–73.16; second‐dose cohort: IRR = 29.61, 95% CI 4.04–217.07). A pictorial summary of major findings in our study and the proposed pathogenesis of myocarditis and pericarditis after COVID‐19 vaccination in children and adolescents is presented in Figure 2.

## 4 DISCUSSIONS

This systematic review analyzed and synthesized evidence from case reports, case series, and original articles regarding the development of myocarditis and pericarditis following COVID‐19 vaccination in children and adolescents. The incidence of myocarditis and pericarditis was higher among younger males than females and patients receiving a second dose of vaccine were more likely to develop these complications. 97% of the reported cases received Pfizer‐BioNTech (BNT162b2) indicating the association of myocarditis and pericarditis with mRNA vaccines. This greater risk being only reported with Pfizer‐BioNTech may be due to the fact that it is currently the only vaccination that has obtained complete FDA approval for use in adolescents.^30^ Chest pain, myalgias, and headache were the most common presentations.^31^ CMR imaging revealed myocardial inflammation and edema in the majority of patients thus confirming the diagnosis of myocarditis according to Lake Lousie criteria.^32^ Majority of the patients had no significant history and all of the patients had a good recovery and were discharged.

Previously, myocarditis has been reported as a side effect of other vaccines such as the smallpox vaccine.^15^ However, the smallpox vaccine differs from the COVID‐19 vaccine in its composition and induced action. The exact mechanism behind the development of myocarditis and pericarditis following the COVID‐19 vaccine has not been defined yet. Molecular mimicry has been hypothesized to be a possible pathology behind this condition according to which the similarity between the viral component of vaccines and specific human proteins can lead to immune cross‐reactivity.^35^ A second proposed mechanism is immune reactivity, which describes the binding of SARS‐CoV‐2 spike protein encoded by mRNA vaccine with angiotensin‐converting enzyme 2 receptor thus resulting in myocardial damage.^36^ There is a possibility that IgG antibodies produced in response to vaccine‐induced spike protein may cross‐react with myocardial contractile protein. Hajjo et al.^37^ has highlighted the central signaling role of IFN‐gamma and TNF‐alpha in both myocarditis and viral disease maps.

Since most of the cases had a mild presentation, the diagnosis was made on the basis of edema and myocardial inflammation observed in CMR imaging. The presence of edema and decreased ejection fraction are indicators of reversible damage in CMR evaluation of early myocarditis and can hence play a role in determining the prognosis and functional recovery of patients. It also sheds light on the possible underlying mechanism and etiology behind this condition. CMR findings revealed a close temporal relationship between a clinical and CMR picture of myocarditis and vaccination; however, these observations alone are not enough to prove whether COVID‐19 immunization caused myocarditis or not. Only large‐scale studies with advanced imaging techniques can provide any possible causative association.^38^

The increased prevalence among males can be related to the differences in hormone signaling hence indicating its involvement in the pathophysiology of COVID‐19 vaccine‐related myocarditis.^39^ There is a decrease in cell‐mediated immune response in females owing to the inhibitory effect of estrogen on pro‐inflammatory T‐cells, whereas, in males, testosterone stimulates a more aggressive T‐helper‐1 cell type immune response by inhibiting anti‐inflammatory cells.^40^ The investigations regarding gender and age disparities in postvaccine myocarditis have revealed that the levels of pro‐inflammatory cytokines such as TNF‐alpha and IFN‐gamma fluctuate between men and women during puberty and then decline later in life, implying hormonal impacts. This corresponds to the findings of the increased incidence of postvaccine myocarditis in adolescents and young adults.^37^

The most important considerations for parents to have confidence in getting their children immunized are vaccine efficacy and safety. The reporting of myocarditis and pericarditis in children and adolescents in large clinical trials of vaccines has remained low.^16^ This can be attributed to the fact that there was limited recruitment from this age group in the clinical trials and also the rarity of this condition in this age group. The Phase 2/3 clinical trial of Pfizer included only 2260 from 12 to 15 years and 754 participants from the age group 16 to 17 years as compared to adults (43 661 participants enrolled in Phase 3 trials).^41^, ^42^

NSAIDs, colchicine, and IVIG remained the most frequently opted treatments for myocarditis and pericarditis following COVID‐19 vaccination which aligns with the current guidelines for the management of viral myocarditis.^43^ Because the pathophysiology of cardiac dysfunction in myocarditis is caused by a maladaptive hyperimmune response driven by a viral infection, medication aimed at modifying the immune response has been proposed as a possible treatment option, and the same treatment plan has been opted for COVID‐19 vaccine‐associated myocarditis. The majority of the patients presented with ST‐elevation, CRP, and troponin levels raised and preserved ventricular ejection fraction thus indicating a mild presentation of this condition.

Though the published literature emphasizes a possible association of COVID‐19 vaccine and myocarditis, the incidence is too small to provide a causal association. Based on available data, the short time span between vaccine administration and development of myocarditis and pericarditis, and the elevated incidence in younger males does suggest a temporal relationship, however, due to the poorly understood mechanism behind this and lack of experimental studies it is difficult to provide a cause‐effect association.^3^, ^44^

Healthcare workers and physicians working with young patients can benefit from the data synthesized in this review and remain updated regarding this association along with its diagnostic modalities and management. Since the majority of the cases were reported after the administration of Pfizer, this raises the concern in the emergency of approval of this vaccine for adolescents and children.

The authors would like to acknowledge a few limitations in the review. Firstly, since there has been no large‐scale clinical trial conducted so far to assess myocarditis/pericarditis associated with COVID‐19 vaccines, this review is based on case reports, case series, and observational studies only. Second, a major proportion of included participants was from original articles, and we did not have access to individual‐level data which imposes another limitation to the devised substantiation. Additionally, we lack sufficient data to support the findings for the population under the age of 12 years. Moreover, due to mild presentation and good recovery, there is a probability that a number of cases might have gone unreported which imposes a limitation in associating the development of myocarditis and pericarditis with the COVID‐19 vaccine. Lastly, a possible publication bias can also exist due to the rarity of this condition.

## 5 CONCLUSION

Myocarditis and pericarditis in children and adolescents after the COVID‐19 vaccine were more prevalent among males and after the second dose of Pfizer. Clinical investigations revealed ST‐segment elevation, CRP, and troponin elevation, and the presence of edema and myocardial damage on CMR imaging. NSAIDs and IVIG were the most commonly opted treatment choices, and all of the cases had a good recovery and were discharged. The cell‐mediated immune responses to vaccination components can cross‐react with heart cells, causing myocardial and pericardial inflammation. However, the exact pathophysiology behind this phenomenon remains unknown. Although the overall incidence is low, however, the clinicians should consider myocarditis and pericarditis as a probable diagnosis when encountering young patients with a history of vaccine administration and presenting with suggestive findings.

## AUTHOR CONTRIBUTIONS

**Maurish Fatima, Muhammad H. A. Khan, and Huzaifa A. Cheema**: Conceptualization; methodology; writing – original draft preparation. **Muhammad S. Ali, Muhammad W. Murad, Aleena Ahmed, and Sarya Swed**: Data extraction; writing; critical appraisal. **Amna Nisar, Aleena Ahmed, and Muhammad Osama**: Data extraction; writing. **Muhammad A. U. Rehman, Hareem Farooq, Usman A. Akbar, and Huzaifa A. Cheema**: Writing – reviewing and editing.

## Supporting information

Supplementary information.

## Data Availability

The data underlying this article are available from the authors on reasonable request.
