# Supplementary material for: Development of myocarditis and pericarditis after COVID‐19 vaccination in children and adolescents: A systematic review
Source: Clin Cardiol. 2023 Jan 2;46(3):243–59. doi: 10.1002/clc.23965 (PMC10018089; doi:10.1002/clc.23965)
Supplement: Supplementary file 1 — Supplementary information. [file CLC-46-243-s001.docx]

Table: S1:

Search strategy for MEDLINE (PubMed format)

| Number | Search terms |
| --- | --- |
| #1 | sars-cov-2 [All Fields] |
| #2 | “sars-cov-2” [mh] |
| #3 | covid [All Fields] |
| #4 | covid-19 [All Fields] |
| #5 | “covid-19” [mh] |
| #6 | coronavirus [All Fields] |
| #7 | "coronavirus" [mh] |
| #8 | #1 OR #2 OR #3 OR #4 OR #5 OR #6 OR #7 |
| #9 | vaccine [All Fields] |
| #10 | "vaccines" [mh] |
| #11 | "vaccination" [mh] |
| #12 | #9 OR #10 OR #11 |
| #13 | #8 AND #12 |
| #14 | "COVID-19 Vaccines/adverse effects" [mh] |
| #15 | #13 OR #14 |
| #16 | myocarditis [All Fields] |
| #17 | “myocarditis” [mh] |
| #18 | pericarditis [All Fields] |
| #19 | "pericarditis" [mh] |
| #20 | #16 OR #17 OR #18 OR #19 |
| #21 | #15 AND #20 |

Table_S2: Quality Appraisal of Case Series

| Sr No | Clear criteria for inclusion | Condition measured in standard and reliable way | Usage of valid methods for identification of condition | Consecutive inclusion of participants | Clear reporting of demographics | Clear reporting of outcomes or follow ups | Clear reporting of presenting site/clinics demographic information | Appropriate statistical analysis | Score | Inclusion/Exclusion | Additional Comment |
| --- | --- | --- | --- | --- | --- | --- | --- | --- | --- | --- | --- |
| Marshal et tal,2021 | 1 | 1 | 1 | 1 | 1 | 1 | 1 | 1 | 8 | Included | None |
| Dionne et al . 2021 | 1 | 1 | 1 | 1 | 1 | 1 | 1 | 0 | 7 | Included | None |
| Dickey et al (2021) | 1 | 1 | 1 | 1 | 1 | 1 | 1 | 1 | 8 | Included | None |
| King et al (2021) | 1 | 1 | 1 | 1 | 1 | 1 | 1 | 0 | 7 | Included | None |
| Park et al 2021 | 1 | 1 | 1 | 1 | 1 | 1 | 1 | 0 | 7 | Included | None |

Table_S3: Quality Appraisal of Case Reports:

| Sr No | Clear Description of Demographic characteristics | Clear Description of history and presentation as a timeline | Clear Description of current clinical condition of patient | Clear Description of diagnostic tests or assessment methods | Clear description of intervention or treatment procedure | Clear description of post-intervention clinical condition | Identification of Adverse events or unanticipated events | Take way lessons | Score | Inclusion/Exclusion | Additional Comments |
| --- | --- | --- | --- | --- | --- | --- | --- | --- | --- | --- | --- |
| Minocha et tal 2021 | 1 | 1 | 1 | 1 | 1 | 1 | 1 | 1 | 8 | Included | None |
| Isaak et al (2021) | 1 | 1 | 1 | 1 | 0 | 0 | 1 | 1 | 6 | Included | No treatment protocol mentioned |
| Watkins et al (2021) | 1 | 1 | 1 | 1 | 1 | 1 | 1 | 1 | 8 | Included | None |

Table_S4: Quality Appraisal of Original Articles:

|  | **Selection** |  |  |  | **Comparability** | **Outcome** |  |  | **Quality** |
| --- | --- | --- | --- | --- | --- | --- | --- | --- | --- |
| Study | Representation of exposed cohort | Selection of non-exposed cohort | Ascertainment of exposure | Demonstration that outcome of interest was not present at start of study | Comparability of cohorts on the basis of design and analysis | Assessment of Outcome | Was follow-up long enough for outcomes to occur | Adequecy of follow-up of cohorts |  |
| Chua *et al,* 2022 | ✰ | 0 | ✰ | ✰ | 0 | ✰ | ✰ | ✰ | Moderate |
| Oster *et al,* 2022 | ✰ | 0 | ✰ | ✰ | 0 | ✰ | ✰ | ✰ | Moderate |
| Nygaard *al*, 2022 | ✰ | 0 | ✰ | ✰ | 0 | ✰ | ✰ | ✰ | Moderate |
| Lai *et al,*2022 | ✰ | ✰ | ✰ | ✰ | ✰✰ | ✰ | ✰ | ✰ | Good |
| Witberg et al, 2021 | ✰ | 0 | ✰ | ✰ | 0 | ✰ | ✰ | ✰ | Moderate |
| Mevorach et al, 2022 | ✰ | 0 | ✰ | ✰ | 0 | ✰ | ✰ | ✰ | Moderate |
